# EGFR-Targeted Pentacyclic Triterpene Analogues for Glioma Therapy

**DOI:** 10.3390/ijms222010945

**Published:** 2021-10-11

**Authors:** Halil I. Ciftci, Mohamed O. Radwan, Belgin Sever, Ahmed K. Hamdy, Safiye Emirdağ, N. Gokce Ulusoy, Ece Sozer, Mustafa Can, Nurettin Yayli, Norie Araki, Hiroshi Tateishi, Masami Otsuka, Mikako Fujita, Mehlika Dilek Altintop

**Affiliations:** 1 Department of Drug Discovery, Science Farm Ltd., Kumamoto 862-0976, Japan; hiciftci@kumamoto-u.ac.jp (H.I.C.); motsuka@gpo.kumamoto-u.ac.jp (M.O.); 2 Medicinal and Biological Chemistry Science Farm Joint Research Laboratory, Faculty of Life Sciences, Kumamoto University, Kumamoto 862-0973, Japan; mohamedradwan@kumamoto-u.ac.jp (M.O.R.); belginsever@anadolu.edu.tr (B.S.); ahmed_alrian88@aun.edu.eg (A.K.H.); mustafacan80@yahoo.com (M.C.); htateishi@kumamoto-u.ac.jp (H.T.); 3 Chemistry of Natural Compounds Department, Pharmaceutical and Drug Industries Research Division, National Research Centre, Dokki, Cairo 12622, Egypt; 4 Department of Pharmaceutical Chemistry, Faculty of Pharmacy, Anadolu University, Eskisehir 26470, Turkey; 5 Department of Medicinal Chemistry, Faculty of Pharmacy, Assiut University, Assiut 71526, Egypt; 6 Chemistry Department, Faculty of Science, Ege University, Izmir 35040, Turkey; sfymrt14@gmail.com (S.E.); ng.ulusoy@gmail.com (N.G.U.); ecsozer95@gmail.com (E.S.); 7 Department of Engineering Sciences, Faculty of Engineering and Architecture, Izmir Katip Celebi University, Izmir 35620, Turkey; 8 Faculty of Pharmacy, Karadeniz Technical University, Trabzon 61080, Turkey; yayli@ktu.edu.tr; 9 Department of Tumor Genetics and Biology, Faculty of Life Sciences, Kumamoto University, Kumamoto 860-8556, Japan; nori@gpo.kumamoto-u.ac.jp

**Keywords:** apoptosis, epidermal growth factor receptor, glioblastoma multiforme, gliomas, pentacyclic triterpenes, molecular docking

## Abstract

Glioma, particularly its most malignant form, glioblastoma multiforme (GBM), is the most common and aggressive malignant central nervous system tumor. The drawbacks of the current chemotherapy for GBM have aroused curiosity in the search for targeted therapies. Aberrantly overexpressed epidermal growth factor receptor (EGFR) in GBM results in poor prognosis, low survival rates, poor responses to therapy and recurrence, and therefore EGFR-targeted therapy stands out as a promising approach for the treatment of gliomas. In this context, a series of pentacyclic triterpene analogues were subjected to in vitro and in silico assays, which were conducted to assess their potency as EGFR-targeted anti-glioma agents. In particular, compound **10** was the most potent anti-glioma agent with an IC_50_ value of 5.82 µM towards U251 human glioblastoma cells. Taking into account its low cytotoxicity to peripheral blood mononuclear cells (PBMCs), compound **10** exerts selective antitumor action towards Jurkat human leukemic T-cells. This compound also induced apoptosis and inhibited EGFR with an IC_50_ value of 9.43 µM compared to erlotinib (IC_50_ = 0.06 µM). Based on in vitro and in silico data, compound **10** stands out as a potential orally bioavailable EGFR-targeted anti-glioma agent endowed with the ability to cross the blood–brain barrier (BBB).

## 1. Introduction

Gliomas, which consist of a group of heterogeneous brain tumors originating from three types of glial cells, namely astrocytes, oligodendrocytes, and ependymal cells, account for almost 80% of primary malignant brain tumors in adults [1,2,3,4,5,6]. The incidence and mortality rate associated with gliomas are expected to increase dramatically in the upcoming years, particularly in developing countries [4]. According to the World Health Organization (WHO) grading, gliomas are categorized into four grades (I-IV) [5]. Among these four grades [6], glioblastoma multiforme (GBM; grade IV) is the most common, aggressive, and malignant form of glioma [7,8]. The main features of GBM are rapid proliferation with poor differentiation, diffuse infiltration into normal brain tissues, angiogenesis, tendency to necrosis, resistance to apoptosis, widespread genomic aberrations [8,9], and these features make GBM challenging to treat [8,9,10,11]. Current treatment protocol involves surgical resection followed by concurrent radiotherapy and temozolomide (TMZ) for 6 weeks, then adjuvant TMZ for 6 months [1,8]. Despite tremendous efforts devoted to improving the therapeutic strategies towards GBM, the prognosis for patients with GBM still remains poor [3] and the median survival rates of these patients are very low [8,9]. The risk of recurrence is high since current therapies do not take into account the unique molecular features of different subtypes of glioma [4].

The efficacy of anti-glioma chemotherapy is limited because of poor drug delivery and inherent chemoresistance [3] or acquired chemoresistance (e.g., to TMZ) after initial treatment [12]. Due to unfavorable pharmacokinetics of chemotherapeutic drugs, poor drug delivery across the blood–brain barrier (BBB) and blood–tumor barrier (BTB) prevents them from exerting their therapeutic action properly. The inherent chemoresistance of the brain endothelium and glioma cells, expressing the drug efflux protein *p*-glycoprotein also impairs the therapeutic efficacy [2,3]. Moreover, clinical applications are limited by adverse effects, such as bone marrow suppression, genotoxic, and teratogenic effects [10].

The shortcomings in the current chemotherapy for malignant gliomas, particularly GBM have aroused great interest in the search for targeted therapies acting on specific molecular targets involved in the pathogenesis of gliomas [13,14]. One of the most widely studied targets is the epidermal growth factor receptor (EGFR), also referred to as ErbB-1/HER1. EGFR is a 170 kDa transmembrane glycoprotein that belongs to the ErbB/HER family of receptor tyrosine kinases (RTKs) [15]. EGFR is involved in key signaling pathways responsible for the growth, proliferation, migration, and survival of tumor cells [7]. The overexpression of EGFR and/or its constitutively activated variant EGFRvIII contributes to the pathogenesis of many types of cancer including GBM [16,17]. Aberrant EGFR signaling leads to poor prognosis and low survival rates for GBM patients, poor responses to therapy, and earlier recurrence after treatment [13,17] and therefore EGFR-targeted therapy has emerged as a promising approach for the management of gliomas [12,13,14,15,16,17,18].

Diverse applications of natural products trace back thousands of years for the treatment of severe diseases. Among these biologically active phytochemicals, pentacyclic triterpenes (PTs) have attracted a great deal of interest as the most valuable sources of pharmacological agents due to their wide range of biological activities including anticancer, antiviral, anti-inflammatory, antimicrobial, antioxidant, antimalarial, neuro-, and hepatoprotective activities [19,20,21,22]. In particular, the number of patents related to PTs endowed with potent anticancer activity is significantly increasing [19]. PTs and their synthetic analogues have been reported to show selective cytotoxic activity towards a huge diversity of cancer cells [19] including GBM cells [23,24,25]. They exert their antitumor action through multiple mechanisms such as apoptosis, cell cycle arrest, and autophagy triggered by their effects on transforming growth factor-beta (TGF-β) and HER cell surface receptors and the downstream signaling pathways, including phosphoinositide 3-kinase (PI3K)-Akt-mammalian target of rapamycin (mTOR), IkB kinase (IKK)/nuclear factor kappa B (NF-κB), signal transducers and activators of transcription (STAT)-3 pathway and mitogen-activated protein kinase (MAPK) cascades [26,27,28,29,30,31]. The search for antitumor PTs has mainly focused on the most abundant groups of PTs, namely the oleanane- (e.g., oleanolic acid, 18β-glycyrrhetinic acid), ursane- (e.g., ursolic acid, asiatic acid) and lupane- (e.g., betulinic acid) triterpenoids [26,27,28,29,30,31,32,33,34,35,36,37]. According to in vitro and in vivo assays, the effects of antitumor PTs on EGFR signaling are fulfilled on the receptor level. Both natural (ursolic, glycyrrhetinic, and oleanolic acids, lupeol, and dimethyl melaleucate) and semisynthetic PTs were found to inhibit tumor cell growth by decreasing phosphorylation of EGFR leading to suppression of downstream signaling (MAPK, PI3K/Akt/NF-κB and/or STAT) pathways [26]. Based on in silico molecular modeling studies conducted to explain the decrease of EGFR-phosphorylation triggered by PTs, ursolic acid, glycyrrhetinic acid, dimethyl melaleucate, some synthetic derivatives of glycyrrhetinic acid and glycyrrhizin are capable of binding to the EGFR tyrosine kinase domain [26]. Some PTs (e.g., oleanolic acid) also decrease EGFR protein expression in some different cancer types [26].

**Figure 1:**
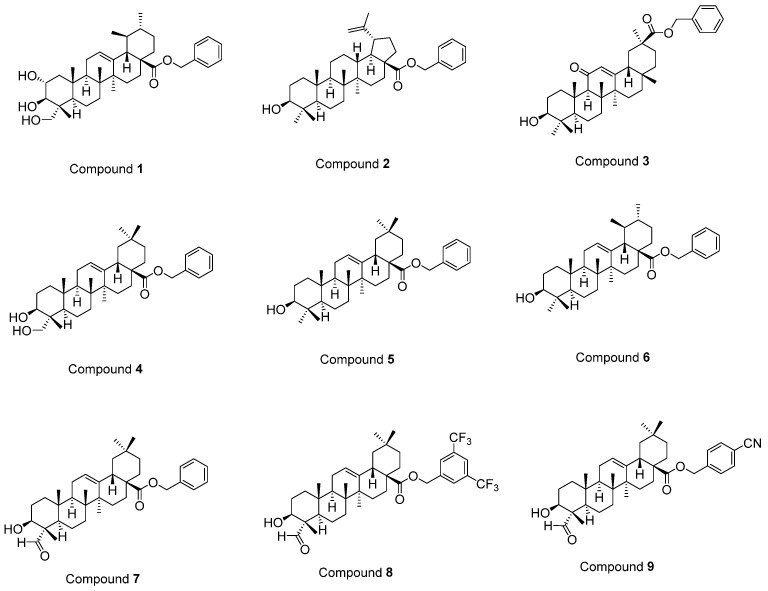
The structures of compounds **1–9** [37].

Previously, we performed in vitro and in silico studies for the benzyl esters of asiatic acid (**1**), betulinic acid (**2**), glycyrrhetinic acid (**3**), hederagenin (**4**), oleanolic acid (**5**), ursolic acid (**6**), and gypsogenin (**7**), and the substituted benzyl esters of gypsogenin (**8, 9**) (Figure 1) to identify anti-chronic myelogenous leukemia (CML) agents targeting ABL1 kinase [37]. A batch of outstanding publications related to the potent antitumor activity of PTs through diverse mechanisms (e.g., EGFR signaling) [19,20,21,22,23,24,25,26,27,28,29,30,31,32,33,34,35,36,37] prompted us to identify EGFR-targeted PTs for the treatment of GBM. In this context, the synthesis of new PTs (**10–13**) was performed efficiently and in vitro and in silico studies were conducted to assess the potential of compounds **1–13** as EGFR-targeted anti-glioma agents.

## 2. Results

**Figure 2:**
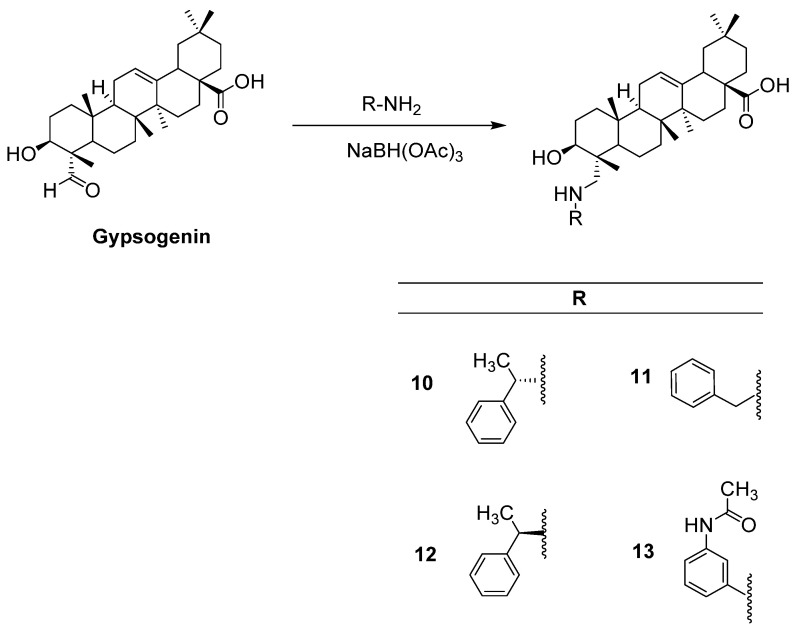
The synthetic route for the preparation of new compounds (**10–13**).

Compounds **1–6**, **8,** and **9** were synthesized as described before [37], whereas compound **7** was prepared according to a previous study [38]. All spectral data were in agreement with those reported. Herein, we report the first reductive amination of a PT, gypsogenin, being endowed with a unique carbaldehyde group. The latter was aminated by four different aromatic amines in the presence of sodium triacetoxyborohydride (Figure 2).

**Figure 3:**
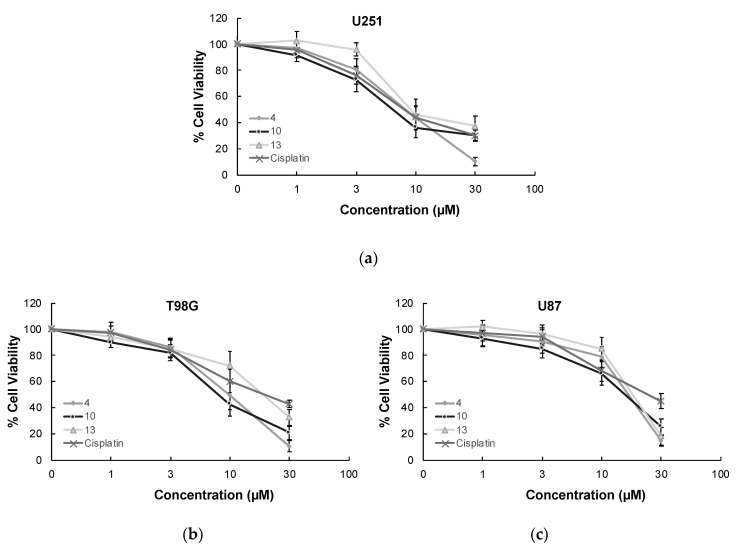

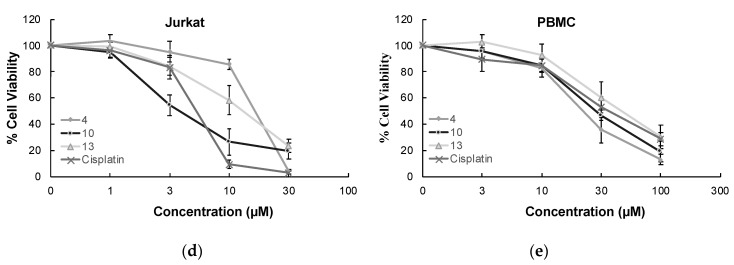
The anticancer effects of compounds **4, 10, 13,** and cisplatin at varying concentrations on U251 cells (**a**), T98G cells (**b**), U87 cells (**c**), Jurkat cells (**d**), and PBMCs (**e**). All descriptive data were expressed as the mean ± standard deviation (SD). All experiments were repeated three times.

MTT assay, the most widely used colorimetric assay for in vitro drug screening [3], was conducted to assess the cytotoxic effects of compounds **1–13** and cisplatin (positive control) on U251, T98G, and U87 human glioblastoma cell lines. As indicated in Table 1, compound **10** was identified as the most effective anti-glioma agent in this series. This compound exerted antitumor action towards U251, T98G, and U87 cells with IC_50_ values of 5.82 µM, 8.19 µM, and 17.04 µM, respectively superior to cisplatin (IC_50_ = 7.70 µM, 16.92 µM, and 20.90 µM, respectively). The potency order of cytotoxic effects of the other compounds on U251 cells was determined as compound **4** > compound **13** > compound **1** > compound **9** > compound **6** > compound **7** > compound **2**. In accordance with the data recorded for U251 cells, compounds **4**, **13,** and **1** followed the same order for T98G and U87 cell lines. The IC_50_ values of compounds **4**, **13,** and **1** for U251 cells were detected as 8.06 µM, 9.95 µM, and 13.18 µM, whereas their IC_50_ values for T98G cells were found as 9.86 µM, 20.19 µM, and 20.54 µM, respectively. Compounds **4**, **13,** and **1** exhibited anticancer activity against U87 cells with IC_50_ values of 19.54 µM, 21.71 µM, and 22.64 µM, respectively. As depicted in Figure 3, compounds **4**, **10,** and **13** showed pronounced antiproliferative effects on U251, T98G, and U87 cells compared to cisplatin at varying concentrations. On the other hand, compounds **3**, **5**, **8**, **11,** and **12** displayed no significant anticancer activity against all tested GBM cell lines at 100 µM concentration.

In order to determine the selectivity of the mode of anti-glioma action, the most effective anticancer agents (compounds **4, 10,** and **13**) were further screened for their cytotoxic effects on Jurkat human leukemic T-cells and human peripheral blood mononuclear cells (PBMCs). The selectivity of compound **10** was found as the most promising between Jurkat cells and PBMCs with a selectivity index (SI) value of 7.90. The anticancer effects of compounds **4**, **10,** and **13** compared to cisplatin at varying concentrations on Jurkat cells and PBMCs also supported this outcome (Figure 3).

**Figure 4:**
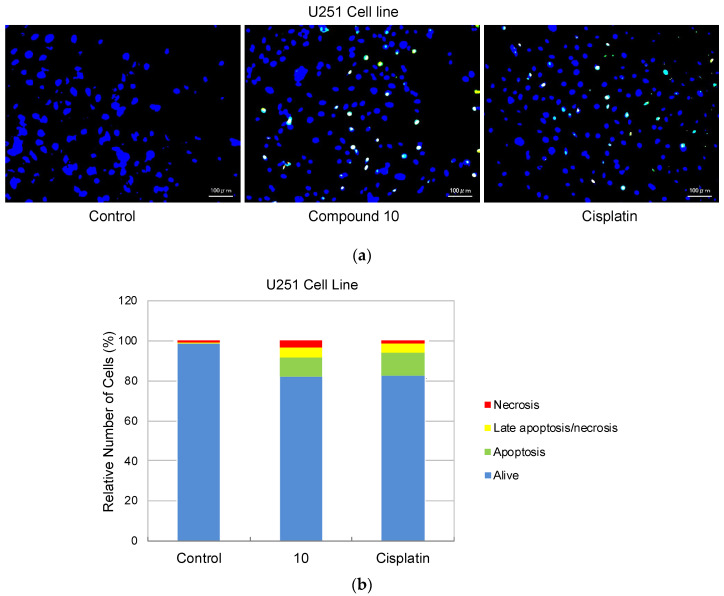

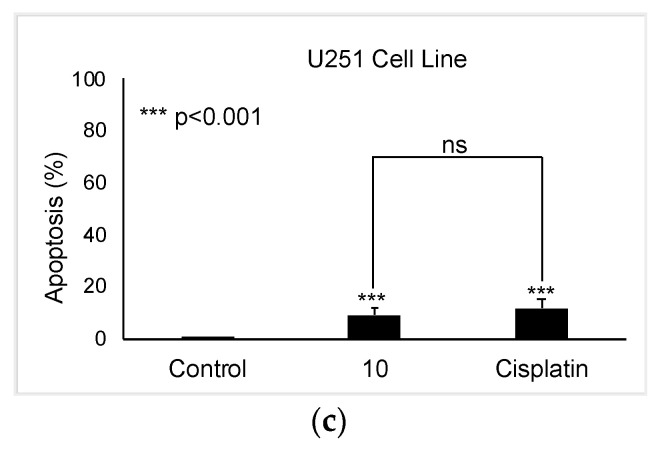
Alteration of U251 cells following exposure to IC_50_ concentration of the control (DMSO), compound **10,** and cisplatin (**a**) for 24 h. The percentage of alive (blue), apoptotic (green), necrotic or late apoptotic (both green and red), and necrotic (red) cells (**b**) was determined by analyzing 100 randomly chosen stained cells in each experiment. Quantification of apoptotic effects of compound **10** and cisplatin (**c**). Data from three independent experiments were expressed as mean ± standard deviation and *p* values were determined using the student’s test.

As compound **10** was designated as the most potent and selective anti-glioma agent according to MTT results, its further apoptotic and EGFR inhibitory effects were also investigated to provide mechanistic insight. Using the annexin V/ethidium homodimer III staining method, the apoptotic effects of compound **10** on U251 cells were evaluated. This method indicates apoptosis and necrosis based on staining green and red, respectively. The results indicated that compound **10** boosted apoptosis in U251 cells with 10.29% similar to cisplatin (13.83%) (Figure 4).

**Figure 5:**
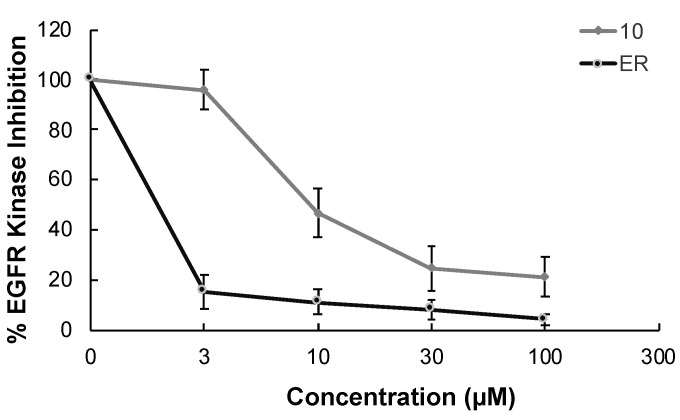
The EGFR kinase inhibition of compound **10** and erlotinib at different concentrations. All descriptive data were expressed as the mean ± SD. All experiments were repeated three times.

**Figure 6:**
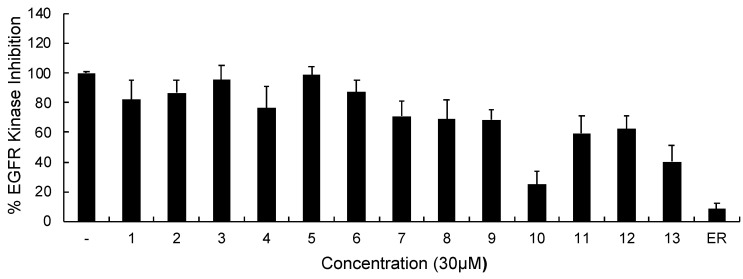
The EGFR kinase inhibition of compounds **1–13** and erlotinib at 30 μM concentration. All descriptive data were expressed as the mean ± SD. All experiments were repeated three times.

**Figure 7:**
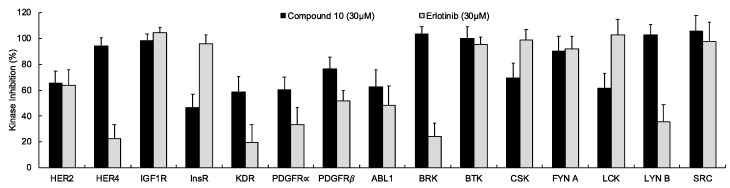
The inhibition of a panel of tyrosine kinases by compound **10** and erlotinib at 30 µM concentration. All descriptive data were expressed as the mean ± SD. All experiments were repeated three times.

In continuation of our mechanistic research, the inhibitory effects of compound **10** on EGFR were analyzed due to the correlation between diminished EGFR signaling with increased anti-glioma activity. It was observed that compound **10** significantly inhibited EGFR with an IC_50_ value of 9.43 µM compared to erlotinib (IC_50_ = 0.06 µM), a first-generation EGFR tyrosine kinase inhibitor (Figure 5). Moreover, Figure 6 also highlighted the significant EGFR activity of compound **10** at 30 µM concentration compared to erlotinib. This outcome also pointed out that newly synthesized compounds displayed higher EGFR inhibition than our previously synthesized compounds. In order to explore the kinase selectivity profiling of compound **10**, the inhibition of compound **10** at 30 µM concentration was examined on a large panel of tyrosine kinase enzymes including TK-1 (HER2, HER4, IGF1R, InsR, KDR, PDGFR-α, and PDGFR-β) and TK-2 (ABL1, BRK, BTK, CSK, FYN A, LCK, LYN B, and SRC) compared to erlotinib. Compound **10** showed the most potent inhibitory activity on the InsR followed by KDR, PDGFR-α, LCK, ABL1, HER2, CSK, PDGFR-β, and FYN A. This compound displayed no significant inhibition against HER4, IGF1R, BTK, BRK, LYN B, and SRC. Compound **10** and erlotinib exhibited similar and moderate HER2, ABL1, and FYN A inhibition, whereas compound **10** showed inhibitory effects on InsR, CSK, and LCK stronger than erlotinib. According to the results, compound **10** revealed a different kinase inhibitory profile than erlotinib as depicted in Figure 7. It can be concluded that compound **10** at 30 µM concentration exhibited the most selective inhibition against EGFR (approximately 2-fold stronger inhibition than InsR, the second promising tyrosine kinase target of compound **10**) among all the tested tyrosine kinases.

**Figure 8:**
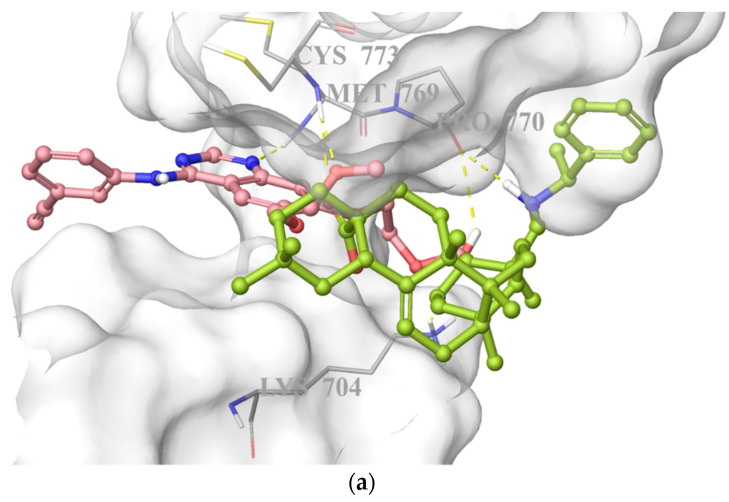

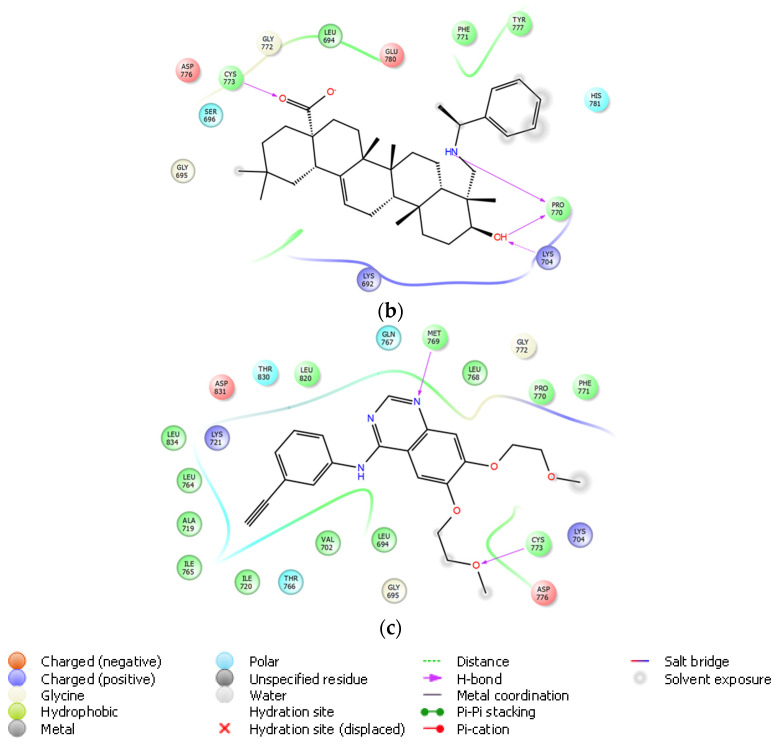
Docking poses of compound **10** and erlotinib (**a**) and docking interactions of compound **10** (**b**) and erlotinib (**c**) in the ATP binding site of EGFR (PDB code: 4HJO). Yellow dashes: hydrogen bonding. Compound **10** and erlotinib were colored in yellow green, and pink, respectively.

On the basis of its significant in vitro EGFR inhibitory potency, molecular docking studies were also carried out to understand the affinity of compound **10** to the adenosine triphosphate (ATP) binding site of the EGFR, which was acquired from the Protein Data Bank (PDB) server (PDB ID: 4HJO) [39]. Molecular docking data demonstrated that compound **10** presented strong affinity at ionized state with favorable hydrogen bonding with Cys773, Pro770, and Lys704 by means of carboxylic acid, (phenylethyl)amino group, and hydroxyl substituent, respectively. This strong affinity could also be attributed to its interaction with Cys773 similar to erlotinib. On the other hand, its less in vitro EGFR inhibitory activity compared to that of erlotinib could be explained by the lack of key interaction with Met769 (Figure 8).

As compared to costly and time-consuming absorption, distribution, metabolism, excretion (ADME) experimental procedures [40], computational models are advantageous approaches to provide access to a set of rapid, yet robust predictive models for physicochemical, and pharmacokinetic properties [10]. In this direction, we performed in silico predictions of some pharmacokinetic parameters of the compounds by the QikProp, a predictive ADME module within the Maestro suite produced by Schrödinger. As depicted in Table 2, the brain/blood partition coefficient (QPlogBB) values of compounds **1–13** ranging from −1.175 to −0.029 were found within the specified limits (−3 to 1.2). The central nervous system (CNS) activity values of compounds **1–13** (−2 to 0) were in agreement within the range (−2 to 2). The QPlogPo/w value, which is a crucial parameter for membrane permeability, metabolism, bioavailability, the toxicity of molecules, and a ligand binding to the receptor [41], was determined as 5.744, 5.453, and 5.745 for compounds **10**, **11,** and **12**, respectively within the specified range (−2 to 6.5). The QPlogPo/w values of other compounds were detected out of limit. Taking into account the importance of hydrogen bonding with pivotal residues in the ATP binding site of EGFR, the number of donor (nHBD) and acceptor (nHBA) sites for hydrogen bonds were calculated. Appropriate nHBD (0 to 4) and nHBA (3.7 to 7.2) values of all compounds within the limits (0 to 6 and 2 to 20, respectively) also supported the outcomes of the molecular docking study. Solvent accessible surface area (SASA) is defined as the accessibility of the residue to the solvent; either it is between lipid or water accessibility and it is also essential to BBB permeability [42]. The SASA values of all compounds were in an optimal range of the specified values (300 to 1000). Compounds **1–13** violated two parameters of Lipinski’s rule of five (maximum is four) and one parameter of Jorgensen’s rule of three (maximum is three).

## 3. Discussion

According to in vitro and in vivo assays, PTs show marked anticancer activity towards a variety of tumor cells through multiple mechanisms [26]. Among PTs, asiatic acid was found to be effective on GBM cells [23,24]. Kavitha et al. treated LN18 and U87-MG human glioma cells with asiatic acid at 20 µM dose for 24 h and pointed out the inhibitory potency of asiatic acid on human glioma cell-induced angiogenesis in vitro [23]. Asiatic acid also decreased vascular endothelial growth factor (VEGF) level (both cellular and secreted) in GBM cells and significantly inhibited VEGF-stimulated angiogenesis in vivo [23]. Garanti et al. demonstrated the antitumor efficacy of asiatic acid as well as asiatic acid-loaded solid lipid nanoparticles (SLNs) on U87-MG cells for 24, 48, and 72 h [24]. Asiatic acid-loaded glyceryl monostearate-SLNs also showed the concentration-dependent apoptotic activity towards U87-MG cells confirming their potential for glioma therapy [24]. In a previous study reported by Wang et al., ursolic acid was found to inhibit U251 cell proliferation via the induction of apoptosis in a dose- and time-dependent manner through TGF-β1/microRNA-21 (miR-21)/programmed cell death 4 (PDCD4) pathway [25].

In a previous study related to the EGFR inhibition by PTs, Sathya et al. pointed out the ability of dimethyl melaleucate to exhibit cell cycle arrest at G0/G1 phase by down-regulation of cyclin D1 through PI3K/Akt inhibition [28]. Dimethyl melaleucate and three structurally related PTs, ursolic acid, 18α-glycyrrhetinic acid, and carbenoxolone suppress EGF mediated breast cancer proliferation through sustained inhibition of EGFR and its downstream effectors STAT3 and cyclin D1 in breast cancer lines [28].

In another study, Chen et al. identified a novel hederagenin−NO donor hybrid, which suppresses the proliferation of gefitinib-resistant H1975 (IC_50_ = 8.1 μM) and osimertinib-resistant H1975-LTC (IC_50_ = 7.6 μM) non-small-cell lung cancer cells via the inhibition of EGFR-LTC kinase (IC_50_ = 0.01 μM), a mutant EGFR kinase [43].

Previously, we reported the synthetic method and the spectral data of the benzyl esters of asiatic acid (**1**), betulinic acid (**2**), glycyrrhetinic acid (**3**), hederagenin (**4**), oleanolic acid (**5**), ursolic acid (**6**), and gypsogenin (**7**), and the substituted benzyl esters of gypsogenin (**8**, **9**) and evaluated their potency as anti-CML agents. Compounds **5**, **8,** and **9** showed marked cytotoxic activity against the K562 CML cell line with IC_50_ values of 5.46, 4.78, and 3.19 µM, respectively as compared to imatinib (IC_50_ = 5.49 µM). Compound **9** was also more effective than compounds **5**, **8,** and imatinib on other leukemic (HL-60, MT-2, and Jurkat), HeLa human cervical carcinoma, MCF-7 human breast adenocarcinoma, and A549 human lung adenocarcinoma cell lines. Compound **8** significantly induced apoptosis (24%) in K562 cells. Compound **5** was found to be the most ABL1 kinase inhibitor in this series with an IC_50_ value of 1.44 µM. In the continuation of this work, herein we designed four new gypsogenin-derived agents through the replacement of the aldehyde group of gypsogenin with the secondary amine group. Encouraged by the scientific reports related to PTs exerting marked antitumor action through different mechanisms (e.g., EGFR signaling) [19,20,21,22,23,24,25,26,27,28,29,30,31,32,33,34,35,36,37], in vitro and in silico assays were conducted to determine their potency as EGFR-targeted anti-glioma agents. Compounds **1–9** were also subjected to in vitro studies to assess their potential for targeted therapy of GBM and compare their biological data with those of compounds **1–4**.

To the best of our knowledge, this is the first study reporting a gypsogenin-derived anti-glioma agent (compound **10**) exerting cytotoxic activity through the induction of apoptosis and the inhibition of EGFR. Compound **10** was found as the most potent anti-glioma agent towards U251, T98G and U87 cells in this series followed by compounds **4** and **13**. In particular, U251 cells were detected more sensitive to these agents than T98G and U87 cells.

The in vitro cytotoxicity (MTT) assay pointed out the crucial role of the three-dimensional spatial arrangement of atoms in anti-glioma activity. Compounds **10** and **12** are epimers, but compound **12** did not show any cytotoxic activity against U251, T98G and U87 cells at the tested concentrations (IC_50_ > 100 µM).

Interestingly, compounds **5** and **8**, which were reported as effective anti-CML agents by our research team [37], did not possess any anticancer activity against U251, T98G and U87 cell lines (IC_50_ > 100 µM). The replacement of the hydroxymethyl group of compound **4** with the methyl group of compound **5** led to the loss of anti-glioma activity. On the other hand, the 3,5-bis(trifluoromethyl) substitution of the benzyl moiety belonging to compound **7** resulted in the formation of compound **8** and, correspondingly, the loss of anti-glioma efficacy.

In vitro studies reveal that compound **10** exerts anti-glioma action through the induction of apoptosis and the inhibition of EGFR. Compound **10** also showed potent inhibitory effects on other tested kinases, particularly InsR. It is important to note that compound **10** possesses a different kinase profile as compared to erlotinib.

Molecular docking studies performed by Sathya et al. [28] for dimethyl melaleucate and three structurally related PTs, ursolic acid, 18α-glycyrrhetinic acid, and carbenoxolone revealed their binding abilities to the ATP binding pocket of EGFR. PTs were found to be capable of forming hydrogen bonds with key amino acid residues, including lysine and cysteine in the binding site of EGFR. 18α-Glycyrrhetinic acid presented important hydrogen bonding with lysine, whereas dimethyl melaleucate was able to form hydrogen bonds with lysine and cysteine residues. On the other hand, carbenoxolone was capable of forming hydrogen bonds in the kinase domain just outside of the ATP binding site. Interestingly, ursolic acid bound to the kinase domain without forming any hydrogen bonds [28].

Chrobak et al. [44] synthesized a new series of 3-phosphate derivatives of betulin carrying different substituents at the C28 position and evaluated their antiproliferative effects on different human cancer cell lines. In order to enlighten the possible mechanism of the most effective anticancer agents, molecular docking was performed in the binding sites of potential anticancer targets described for the various triterpene derivatives, including EGFR. Among these derivatives, 3-diethoxyphosphoryl-28-propynoylbetulin (**4**) formed interactions with Cys773 and some important residues. In another study conducted by the same group [45], 30-diethylphosphate derivatives of betulin were synthesized and investigated for their in vitro cytotoxic activity against diverse human cancer cell lines, including SNB-19 human glioma cell line. The most potent compounds, 30-diethoxyphosphoryloxy-28-propynoylbetulin (**7a**) and 28–(2-butynoyl)-30-diethoxyphosphoryloxybetulin (**7b**), were further screened for their apoptotic effects. The most active products were docked to the active site of the EGFR. The results showed that compounds **7a** and **7b** displayed hydrophobic interactions and hydrogen bonds with lysine, cysteine, and threonine residues in the binding site of EGFR.

The molecular docking analysis in this study put emphasis on the key interactions of compound **10** with crucial residues (lysine and cysteine) in the ATP binding site of EGFR. These findings were found in agreement with in silico results of the previous works carried out by Sathya et al. [28] and Chrobak et al. [44,45]. Intriguingly, all these derivatives missed the essential interaction with Met769, which was proved to play an important role in the binding of erlotinib to the ATP binding site of EGFR. Consequently, diminished in vitro EGFR inhibitory activity of compound **10** compared to erlotinib might be attributed to the lack of this interaction.

The BBB, which is the major impediment in drug delivery to the tumor [46], limits the efficacy of anti-glioma agents [10]. In order to overcome this problem, the discovery of anti-glioma agents endowed with a favorable pharmacokinetic profile and bioavailability along with the ability to cross the BBB is of great importance and, therefore ADME properties are critical features for anti-glioma agents to consider [3]. Chrobak et al. [45] also reported in silico ADME parameters of 30-diethylphosphate derivatives of betulin. Among these parameters, their nHBA, nHBD, and tPSA values demonstrated their oral bioavailability. The log BB values of some triterpenes were within an acceptable range for drug-likeness (log BB −3 to 1.2). The in silico prediction of our current paper was compatible with the aforementioned study. The optimum QPlogBB, QPlogPo/w, nHBA, nHBD, and SASA values of compound **10** accentuated its favorable pharmacokinetic profile. The compliance of compound **10** with Lipinski’s rule of five and Jorgensen’s rule of three also supported its good oral bioavailability and drug-like features.

Compound **10** has a different chemical structure from currently available anti-glioma drugs and, thus, it could be helpful in overcoming resistance. In conclusion, compound **10** stands out as a promising lead compound for targeted therapy of GBM.

Taking into account the knowledge obtained from in vitro and in silico assays, further studies can be carried out to design a new series of PTs with improved EGFR inhibitory potency by the structural modification of compound **10** and investigate not only their direct cytotoxic effects on GBM cells but also their effects on the tumor microenvironment (e.g., suppression of angiogenesis, tumor-related inflammation, etc.).

## 4. Materials and Methods

### 4.1. Chemistry

The chemicals were purchased from Sigma-Aldrich (St. Louis, MO, USA), Honeywell Fluka (Morristown, NJ, USA), Kanto Chemical (Tokyo, Japan), Nacalai Tesque (Kyoto, Japan), Tokyo Chemical Industry (Tokyo, Japan), and FUJIFILM Wako (Osaka, Japan). The commercially available reagent-grade chemicals were used without further purification. The reaction progress was monitored by thin-layer chromatography (TLC) on TLC silica gel 60 F_254_ aluminum sheets (Merck, Darmstadt, Germany). The flash column chromatography was performed on Silica Gel 60N (40–100 mesh, Kanto Chemical, Tokyo, Japan). Melting points (mp) were determined on a Yanaco melting point apparatus (Kyoto, Japan) and were uncorrected. ^1^H and ^13^C NMR spectra were recorded on a Bruker Avance 600 spectrometer (600 MHz) (Billerica, MA, USA). Mass spectrometry (MS) and high-resolution mass spectrometry (HRMS) data were recorded on a JEOL (Tokyo, Japan) JMS-DX303HF using positive fast atom bombardment (FAB) technique with 3-nitrobenzyl alcohol as the matrix.

#### General Method of the Preparation of Compounds **10**–**13**

In a dry two-neck flask equipped with a magnet stirrer, (117 mg, 0.25 mmol) of a gypsogenin was dissolved in 5 mL dry 1,2-dichloroethane followed by the addition of the relevant amine (0.25 mmol) and then treated with solid sodium triacetoxyborohydride (73 mg, 0.35 mmol) and the mixture was stirred for 6 h at room temperature. The reaction was then quenched with saturated sodium hydrogen carbonate solution, which was then extracted three times with dichloromethane. The combined organic layers were dried over anhydrous sodium sulfate, and the solvent was removed under reduced pressure. Each product was purified by flash chromatography using the appropriate solvent system.

10-Hydroxy-2,2,6a,6b,9,12a-hexamethyl-9-[(((*R*)-1-phenylethyl)amino)methyl]-1,2,3,4,4a,5,6,6a,6b,7,8,8a,9,10,11,12,12a,12b,13,14b-icosahydropicene-4a-carboxylic acid (**10**): Compound **10** was obtained by the reaction with (*R*)-1-phenylethanamine as a white solid (21 mg, 15%); mp 240–243 °C. ^1^H NMR (600 MHz, DMSO-*d*_6_) δ 7.33–7.29 (m, 4H), 7.23–7.20 (m, 1H), 5.16 (s, 1H), 3.58 (q, *J* = 6.6 Hz, 1H), 2.74 (dd, *J* = 13.3, 3.9 Hz, 1H), 2.58 (d, *J* = 12.0 Hz, 1H), 1.91 (d, *J* = 12.0 Hz, 2H), 1.80 (dd, *J* = 8.6, 2.2 Hz, 2H), 1.67–0.99 (m, 20H), 1.26 (d, *J* = 6.6 Hz, 3H), 1.11 (s, 3H), 0.88 (d, *J* = 2.5 Hz, 6H), 0.85 (s, 3H), 0.68 (s, 3H), 0.63 (s, 3H) (Figure S1). ^13^C NMR (150 MHz, DMSO-*d*_6_) δ 178.41, 146.15, 143.91, 128.12, 126.38, 126.29, 121.54, 74.35, 58.37, 55.77, 49.78, 47.33, 45.80, 45.53, 41.43, 40.99, 40.95, 40.30, 39.04, 37.97, 36.66, 33.45, 32.78, 32.31, 32.16, 30.33, 27.24, 26.37, 25.53, 24.04, 23.39, 22.93, 22.74, 18.03, 16.96, 15.49, 13.41 (Figure S2). MS (FAB) *m/z* 576.5 (M + H)^+^; HRMS (FAB) Calcd. for C_38_H_58_O_3_N: 576.4417. Found: 576.4440.

9-((Benzylamino)methyl)-10-hydroxy-2,2,6a,6b,9,12a-hexamethyl-1,2,3,4,4a,5,6,6a,6b,7,8,8a,9,10,11,12,12a,12b,13,14b-icosahydropicene-4a-carboxylic acid (**11**): Compound **11** was obtained from the reaction with benzyl amine as a white solid (30 mg, 41%); mp 246–249 °C. ^1^H NMR (600 MHz, DMSO-*d*_6_) δ 7.34–7.29 (m, 4H), 7.24–7.21 (m, 1H), 5.19 (s, 1H), 3.78 (d, *J* = 13.6 Hz, 1H), 3.64 (d, *J* = 13.6 Hz, 1H), 3.45 (dd, *J* = 11.0, 4.9 Hz, 1H), 2.77 (dd, *J* = 14.1, 4.1 Hz, 1H), 2.71 (d, *J* = 12.2 Hz, 1H), 2.19 (d, *J* = 12.2 Hz, 1H), 1.12 (s, 3H), 1.01–1.93 (m, 22H) 0.90 (s, 9H), 0.75 (d, *J* = 8.3 Hz, 3H), 0.69 (s, 3H) (Figure S3). ^13^C NMR (150 MHz, DMSO-*d*_6_) δ 178.40, 144.63, 143.91, 128.01, 127.81, 126.46, 121.54, 73.53, 56.50, 53.96, 49.19, 47.31, 45.80, 45.53, 41.44, 41.24, 40.93, 40.29, 39.02, 38.00, 36.63, 33.44, 32.78, 32.24, 32.16, 30.33, 27.27, 26.38, 26.24, 25.58, 23.40, 22.94, 22.74, 17.87, 16.94, 15.55, 13.70 (Figure S4). MS (FAB) *m/z* 562.5 (M + H)^+^; HRMS (FAB) Calcd. for C_37_H_56_O_3_N: 562.4260. Found: 562.4283.

10-Hydroxy-2,2,6a,6b,9,12a-hexamethyl-9-[(((*S*)-1-phenylethyl)amino)methyl]-1,2,3,4,4a,5,6,6a,6b,7,8,8a,9,10,11,12,12a,12b,13,14b-icosahydropicene-4a-carboxylic acid (**12**). Compound **12** was obtained through the reaction with (*S*)-1-phenylethanamine as a white solid (31 mg, 22%); mp 240–243 °C. ^1^H NMR (600 MHz, DMSO-*d*_6_) δ 7.33–7.29 (m, 4H), 7.22–7.20 (m, 1H), 5.15 (s, 1H), 3.58 (q, *J* = 6.6 Hz, 1H), 2.74 (dd, *J* = 13.5, 4.4 Hz, 1H), 2.58 (d, *J* = 12.0 Hz, 1H), 1.91 (d, *J* = 12.0 Hz, 2H), 1.80 (dd, *J* = 7.5, 3.1 Hz, 1H), 1.66–0.99 (m, 20H), 1.26 (d, *J* = 6.6 Hz, 3H), 1.11 (s, 3H), 0.88 (s, 6H), 0.85 (s, 3H), 0.68 (s, 3H), 0.63 (s, 3H) (Figure S5). ^13^C NMR (150 MHz, DMSO-*d*_6_) δ 178.54, 146.33, 143.88, 128.09, 126.55, 126.49, 121.48, 73.42, 58.49, 55.20, 49.05, 47.15, 45.67, 45.41, 41.29, 40.91, 40.74, 40.07, 38.83, 37.77, 36.55, 33.30, 32.82, 32.06, 30.89, 30.37, 27.19, 26.21, 25.65, 24.59, 23.37, 22.87, 22.60, 17.65, 16.86, 15.63, 13.86 (Figure S6). MS (FAB) *m/z* 576.4 (M + H)^+^; HRMS (FAB) Calcd. for C_38_H_58_O_3_N: 576.4417. Found: 576.4422.

9-[((3-Acetamidophenyl)amino)methyl]-10-hydroxy-2,2,6a,6b,9,12a-hexamethyl-1,2,3,4,4a,5,6,6a,6b,7,8,8a,9,10,11,12,12a,12b,13,14b-icosahydropicene-4a-carboxylic acid (**13**). Compound **13** was synthesized using 3′-aminoacetanilide as a white solid (24 mg, 16%); mp 265–267 °C. ^1^H NMR (600 MHz, MeOD) δ: 6.99 (t, *J* = 8.0 Hz, 1H), 6.96 (t, *J* = 2.0 Hz, 1H), 6.71 (ddd, *J* = 8.0, 2.0, 0.8 Hz, 1H), 6.47 (ddd, *J* = 8.0, 2.0, 0.8 Hz, 1H), 5.20 (dd, *J* = 7.6, 3.7 Hz, 1H), 3.53 (dd, *J* = 10.8, 3.7 Hz, 1H), 3.18 (d, *J* = 13.4 Hz, 1H), 2.93 (d, *J* = 13.4 Hz, 1H), 2.86–2.84 (m, 1H), 2.08 (s, 3H), 1.15–1.93 (m, 24H), 1.09 (s, 3H), 0.98 (s, 3H), 0.93 (s, 3H), 0.87 (s, 3H), 0.83 (s, 3H), 0.81 (s, 3H) (Figure S7). ^13^C NMR (150 MHz, MeOD) δ: 180.38, 170.29, 159.10, 148.30, 143.90, 133.91, 129.37, 122.09, 114.21, 109.60, 72.66, 55.35, 48.18, 46.23, 45.85, 41.89, 41.56, 41.33, 39.18, 39.07, 37.86, 36.82, 36.55, 33.51, 32.42, 32.16, 30.20, 27.43, 26.41, 26.04, 25.08, 23.12, 22.65, 22.58, 22.50, 18.22, 16.42, 14.96, 12.25, 11.31 (Figure S8). MS (FAB) *m/z* 605.4 (M + H)^+^; HRMS (FAB) Calcd. for C_38_H_57_O_4_N_2_: 605.4318. Found: 605.4330.

### 4.2. Biochemistry

#### 4.2.1. Cell Culture and Drug Treatment

U251, T98G (Japanese Collection of Research Bioresources Cell Bank), and U87 human glioblastoma cell lines were incubated in Dulbecco’s Modified Eagle Medium (DMEM)/Ham’s F12 (Wako Pure Chemical Industries, Osaka, Japan), Eagle’s Minimum Essential Medium (EMEM; Wako Pure Chemical Industries, Osaka, Japan), and DMEM (Gibco/Thermo Fisher Scientific, Waltham, MA, USA) respectively, and supplemented with 10% fetal bovine serum (FBS; Sigma Aldrich, MO, USA). Jurkat human leukemic T-cell line was cultured in Roswell Park Memorial Institute (RPMI) 1640 (Wako Pure Chemical Industries, Osaka, Japan), supplemented with 10% FBS. PBMCs (Precision Bioservices, Frederick, MD, USA) were incubated in RPMI 1640 and supplemented with 10% human serum AB (HS) (Gemini, Woodland, CA, USA). All media were supplemented with 89 μg/mL streptomycin (Meiji Seika Pharma, Tokyo, Japan) at 37 °C in a humid atmosphere and 5% CO_2_. For each experiment, growing cells were plated at 4 × 10^4^ cells/mL and 6 × 10^5^ cells/mL into 24-well and 96-well microtiter tissue culture plates (Iwaki brand Asahi Glass Co., Chiba, Japan) respectively and incubated for 72 h before the addition of the drugs (the optimal cell number for cytotoxicity assays was determined in preliminary experiments). The stock solutions of the compounds and cisplatin (Sigma-Aldrich, St. Louis, MO, USA) in concentrations between 0.1–10 mM were prepared in dimethyl sulfoxide (DMSO; Wako Pure Chemical Industries, Osaka, Japan) and in H_2_O respectively, then were added to fresh culture medium. The concentration of DMSO and H_2_O in the final culture medium was 1% which had no effect on the cell viability [47].

#### 4.2.2. MTT Assay

The MTT (Dojindo Molecular Technologies, Kumamoto, Japan) assay was carried out using the U251, T98G, U87, Jurkat, and PBMC cells to determine the cytotoxicity of compounds **1–13** and cisplatin as previously described in the literature with small modifications [48,49]. Treated cells were incubated with different concentrations (1–100 µM) of the compounds for 72 h at 37 °C in the CO_2_ incubator. About 100 µL (1.1 mg/mL) of MTT solution was added to each well and after further incubation for 4 h, formazan crystals formed. At the end of this period, the medium was removed and the formazan crystals were solubilized by the addition of 100 μL DMSO to each well. The absorbance was read on an Infinite M1000 plate reader (Tecan, Mannedorf, Switzerland) at a wavelength of 550 nm with background subtraction at 630 nm. All experiments were performed in triplicate and IC_50_ values were defined as the drug concentrations that reduced absorbance to 50% of control values.

#### 4.2.3. Detection of Apoptotic and/or Necrotic Cells

U251 cells (4 × 10^4^ cells/well) were seeded in each well of a 24-well plate with compound **10** and cisplatin at 5 μM concentration for 24 h. Then, the apoptotic/necrotic/healthy cells detection kit protocol was applied according to the manufacturer’s instructions (PromoKine, Heidelberg, Germany) with few modifications [50]. Briefly, the cells were washed twice with 1 × binding buffer and then treated with FITC-Annexin V (5 μL), ethidium homodimer III (5 μL), and Hoechst 33342 (5 μL) for 20 min at room temperature in a protected-light environment. After washing in 1 × binding buffer, cells were analyzed under an all-in-one fluorescence microscope Biorevo Fluorescence BZ-9000 (Keyence, Osaka, Japan). Healthy cells, apoptotic cells, late apoptotic or necrotic cells, and necrotic cells were counted as previously described [51].

#### 4.2.4. Kinase Inhibition Assay

The kinase profiling assay protocol (TK1 and TK-2) was applied according to the manufacturer‘s instructions (Promega Corporation, Madison, WI, USA) with few modifications [52,53,54]. In regard to this protocol, the EGFR, HER2, HER4, IGF1R, InsR, KDR, PDGFR-α, PDGFR-β, ABL1, BRK, BTK, CSK, FYN A, LCK, LYN B, and SRC kinase stocks in the kinase strips were diluted with 95 µL 2.5x kinase buffer and their substrate stock in the substrate strip was diluted with 15 µL of 100 µM ATP solutions. Then, the kinase reactions were performed using 2 µL of the kinase working stocks, 2 µL of the ATP/substrate working stocks, and 1 µL of compound solution (0.1–100 µM) or 5% DMSO solution in the 384-well plate. After 4 h of incubation at room temperature, the activity of kinases was detected using ADP-Glo Kinase Assay (Promega Corporation, Madison, WI, USA) according to the manufacturer‘s protocol. Briefly, 5 µL of ADP-Glo reagent was added into each well of 384-well plate and the mixture was incubated at room temperature for 40 min to stop the kinase reaction and deplete any ATP remaining. Then, 10 µL Kinase Detection Reagent was added and the mixture was incubated for a further 30 min to convert the kinase-produced ADP to ATP, and then to light. The inhibitory kinase activity of the compounds in a dose–response mode was measured by a luminescence plate reader Infinite M1000 (Tecan, Grödig, Austria) and the IC_50_ values of tested compounds required to decrease the kinase activity by 50% were calculated using ImageJ software.

### 4.3. Molecular Docking

The X-ray crystallographic structure of EGFR was obtained from the PDB server (PDB code: 4HJO) [39] and optimized for molecular docking studies in the protein preparation module of Schrödinger software. Optimized Potential Liquid Simulations (OPLS_2005) force field was used at physiological pH in ligand preparation module of Schrödinger software for preparation of compound **10** and erlotinib with energy minimization. Finally, Grid Generation and Glide/XP docking protocols were applied, respectively in molecular docking simulations (Schrödinger Release 2016-2: Schrödinger, LLC, New York, NY, USA).

### 4.4. In Silico ADME Prediction

Some crucial pharmacokinetic properties of compounds **1–13** were speculated using QikProp module of Schrödinger software (Schrödinger Release 2016-2: QikProp, Schrödinger, LLC, New York, NY, 2016).

## 5. Conclusions

Previously, we substantiated the anticancer effects of PTs (**1–9**) associated with apoptotic capability and ABL1 kinase inhibitory potency. In the current study, the rationally designed new PTs (**10–13**) were synthesized through a facile synthetic route. Then, compounds **1–13** were in vitro searched for their anticancer efficacy against U251, T98G, and U87 human glioma cell lines. Compound **10**, the most potent anti-glioma agent in this series, underwent further mechanistic studies, including the determination of apoptotic induction and inhibitory effects on a large panel of tyrosine kinases, particularly EGFR. The molecular docking study performed in the ATP binding site of EGFR also supported the biological data of compound **10** relevant to in vitro EGFR inhibition. In silico pharmacokinetic evaluation indicates that compound **10** is a promising bioavailable anti-glioma drug candidate targeting EGFR for further studies.

## Figures and Tables

**Table 1:** The cytotoxic effects of the compounds on U251, T98G, U87, and Jurkat cells, PBMCs.

Compound	IC_50_ Value (µM)					SI ^1^
	U251 Cells	T98G Cells	U87 Cells	Jurkat Cells	PBMCs	
**1**	13.18 ± 3.19	20.54 ± 4.34	22.64 ± 6.75			
**2**	24.00 ± 4.98	>100	>100			
**3**	>100	>100	>100			
**4**	8.06 ± 2.04	9.86 ± 2.21	19.54 ± 4.52	9.97 ± 3.24	21.91 ± 5.13	2.20
**5**	>100	>100	>100			
**6**	16.68 ± 3.17	64.12 ± 7.36	79.70 ± 10.08			
**7**	17.98 ± 2.23	61.11 ± 5.13	60.93 ± 8.87			
**8**	>100	>100	>100			
**9**	14.13 ± 3.41	56.55 ± 6.08	>100			
**10**	5.82 ± 1.66	8.19 ± 2.42	17.04 ± 4.92	3.56 ± 1.45	28.12 ± 5.05	7.90
**11**	>100	>100	>100			
**12**	>100	>100	>100			
**13**	9.95 ± 2.04	20.19 ± 5.47	21.71 ± 6.09	12.08 ± 1.64	43.15 ± 8.32	3.57
Cisplatin	7.70 ± 2.81	16.92 ± 3.95	20.90 ± 5.16	4.87 ± 2.00	34.67 ± 7.11	7.12

^1^ SI = IC_50_ for PBMCs/IC_50_ for Jurkat cells.

**Table 2:** Predicted ADME properties of compounds **1–13**.

Compound	QPlogBB * (−3 to 1.2)	CNS * (−2 to 2)	QPlogPo/w * (−2 to 6.5)	nHBD * (0 to 6)	nHBA * (2 to 20)	SASA * (300–1000)	Rule of Five **	Rule of Three ***
**1**	−0.595	−1	5.877	3	7.1	743.017	2	1
**2**	−0.388	0	8.081	1	3.7	813.580	2	1
**3**	−0.529	0	7.171	1	5.7	827.146	2	1
**4**	−0.574	0	6.781	2	5.4	756.322	2	1
**5**	−0.120	0	7.783	1	3.7	746.935	2	1
**6**	−0.105	0	7.829	1	3.7	741.337	2	1
**7**	−0.569	0	6.968	0	4.7	811.227	2	1
**8**	−0.035	0	8.795	0	4.7	799.503	2	1
**9**	−1.175	−2	6.275	0	6.2	780.099	2	1
**10**	−0.061	−1	5.744	3	5.2	863.864	2	1
**11**	−0.038	−1	5.453	3	5.2	848.674	2	1
**12**	−0.029	−1	5.745	3	5.2	863.850	2	1
**13**	−1.057	−2	6.866	4	7.2	890.067	2	1

* QPlogBB: Brain/blood partition coefficient, CNS: Predicted central nervous system activity. QPlogPo/w: Predicted octanol/water partition coefficient. nHBD and nHBA: Estimated number of hydrogen bonds that would be donated and accepted, respectively, by the solute to water molecules in an aqueous solution. Values are averages taken over a number of configurations, so they can be non-integer. SASA: Total solvent accessible surface area in square angstroms using a probe with a 1.4 Å radius. ** Rule of Five: Number of violations of Lipinski’s rule of five. *** Rule of Three: Number of violations of Jorgensen’s rule of three.

## Data Availability

Not applicable.

## Supplementary Materials

The following are available online at https://www.mdpi.com/article/10.3390/ijms222010945/s1.
