# Supplementary material for: EGFR-Targeted Pentacyclic Triterpene Analogues for Glioma Therapy
Source: Int J Mol Sci. 2021 Oct 11;22(20):10945. doi: 10.3390/ijms222010945 (PMC8537327; doi:10.3390/ijms222010945)
Supplement: Supplementary file 1 [file ijms-22-10945-s001.zip › ijms-1376516-supplementary.pdf]

Supplementary Material: NMR Data

EGFR-Targeted Pentacyclic Triterpene Analogues for Glioma Therapy

Halil I. Ciftci[1,2,†], Mohamed O. Radwan[2,3,†], Belgin Sever[2,4], Ahmed K. Hamdy[2,5], Safiye Emirdağ[6], N. Gokce Ulusoy[6], Ece Sozer[6], Mustafa Can[2,7], Nurettin Yayli[8], Norie Araki[9], Hiroshi Tateishi[2], Masami Otsuka[1,2], Mikako Fujita[2,*] and Mehlika Dilek Altintop[4,*]

1 Department of Drug Discovery, Science Farm Ltd., 862-0976 Kumamoto, Japan; hiciftci@kumamoto-u.ac.jp (H.I.C.); motsuka@gpo.kumamoto-u.ac.jp (M.O.)
2 Medicinal and Biological Chemistry Science Farm Joint Research Laboratory, Faculty of Life Sciences, Kumamoto University, 862-0973 Kumamoto, Japan; htateishi@kumamoto-u.ac.jp (H.T.); mohamedradwan@kumamoto-u.ac.jp (M.O.R.)
3 Chemistry of Natural Compounds Department, Pharmaceutical and Drug Industries Research Division, National Research Centre, Dokki, 12622 Cairo, Egypt;
4 Department of Pharmaceutical Chemistry, Faculty of Pharmacy, Anadolu University, 26470 Eskisehir, Turkey; belginsever@anadolu.edu.tr (B.S.)
5 Department of Medicinal Chemistry, Faculty of Pharmacy, Assiut University, 71526 Assiut, Egypt; ahmed_alrian88@aun.edu.eg (A.K.H.)
6 Chemistry Department, Faculty of Science, Ege University, 35040 Izmir, Turkey; sfymrt14@gmail.com (S.E.); ng.ulusoy@gmail.com (N.G.U.); ecsozer95@gmail.com (E.S.)
7 Department of Engineering Sciences, Faculty of Engineering and Architecture, Izmir Katip Celebi University, 35620 Izmir, Turkey; mustafacan80@yahoo.com (M.C.)
8 Faculty of Pharmacy, Karadeniz Technical University, 61080 Trabzon, Turkey; yayli@ktu.edu.tr (N.Y.)
9 Department of Tumor Genetics and Biology, Faculty of Life Sciences, Kumamoto University, 860-8556 Kumamoto, Japan; nori@gpo.kumamoto-u.ac.jp (N.A.)

* Correspondence: mfujita@kumamoto-u.ac.jp (M.F.); mdaltintop@anadolu.edu.tr (M.D.A.); Tel.: +90-222-335-0580 (ext. 3807) (M.D.A.)
† These authors contributed equally to this work.

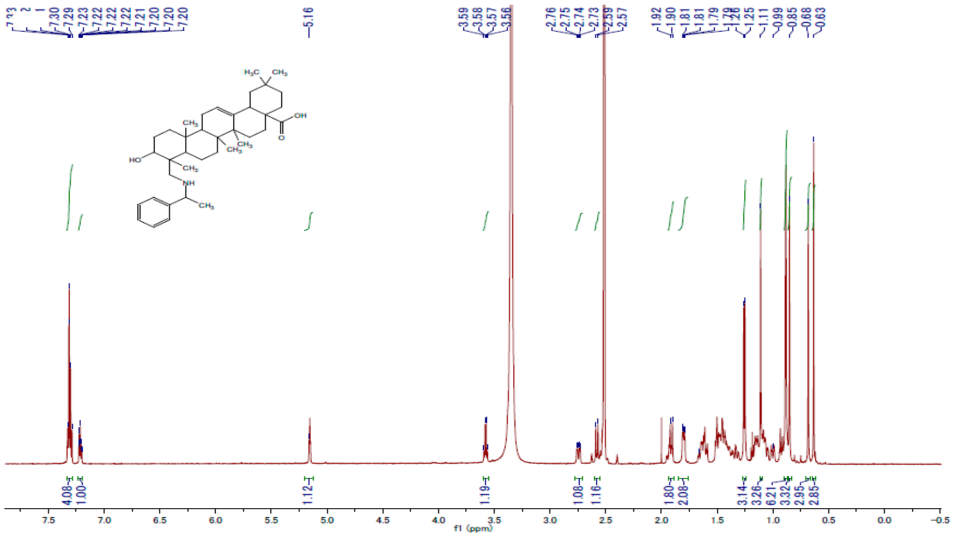

Figure S1: ^{1}H NMR spectrum of compound **10**

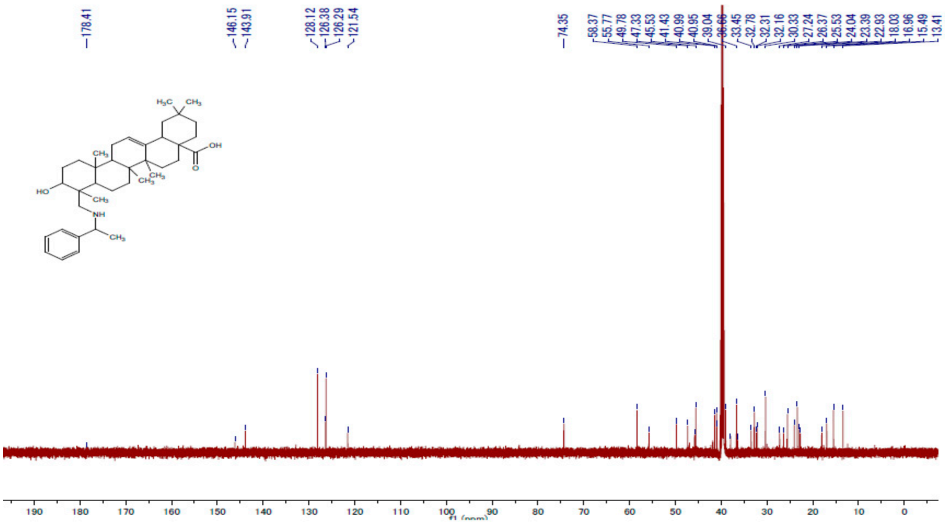

Figure S2: ^{13}C NMR spectrum of compound **10**

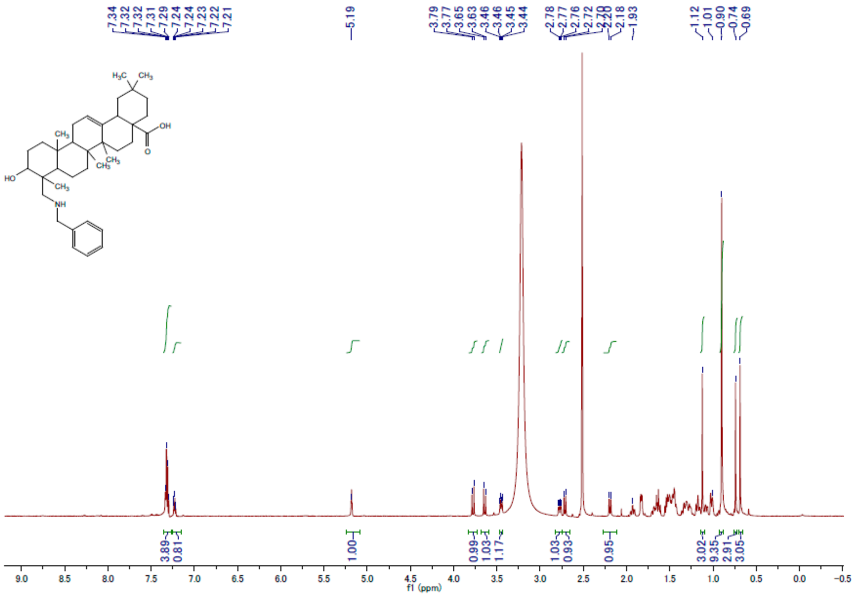

Figure S3: ^{1}H NMR spectrum of compound **11**

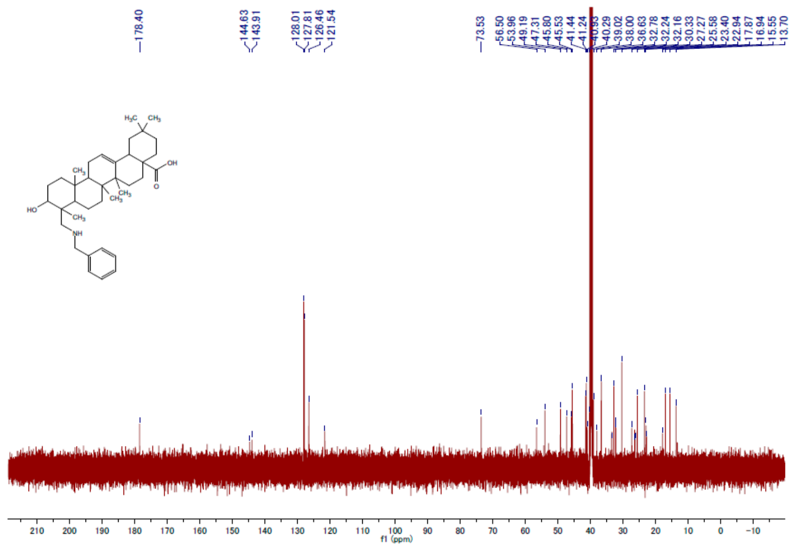

Figure S4: ^{13}C NMR spectrum of compound **11**

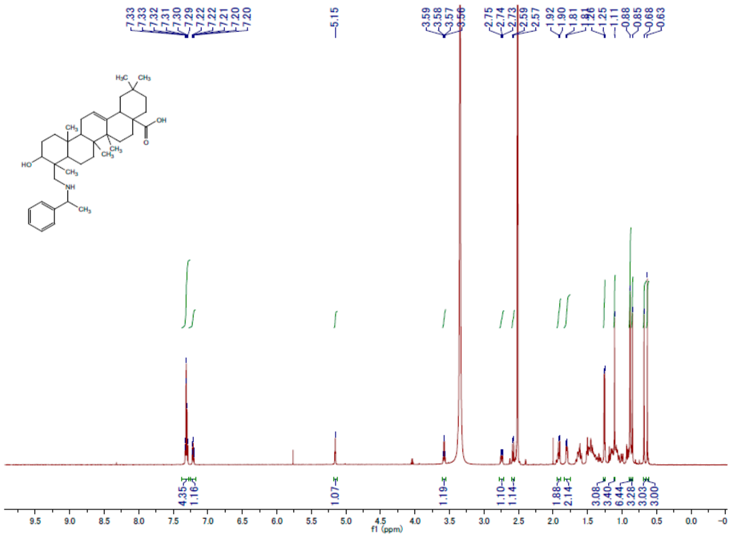

Figure S5: ^{1}H NMR spectrum of compound **12**

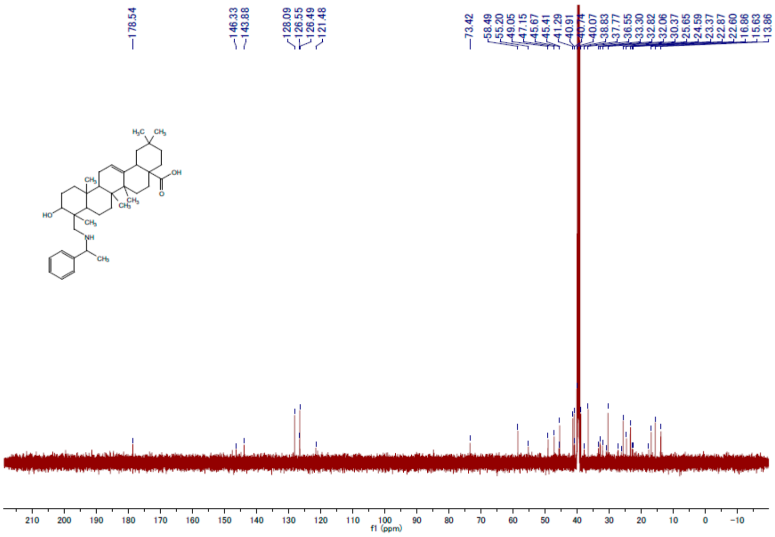

Figure S6: ^{13}C NMR spectrum of compound **12**

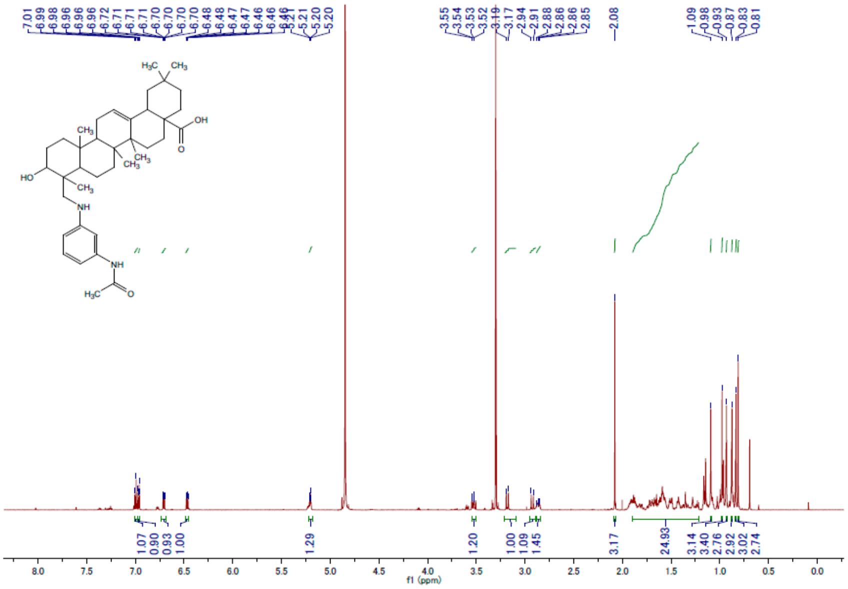

Figure S7: ^{1}H NMR spectrum of compound **13**

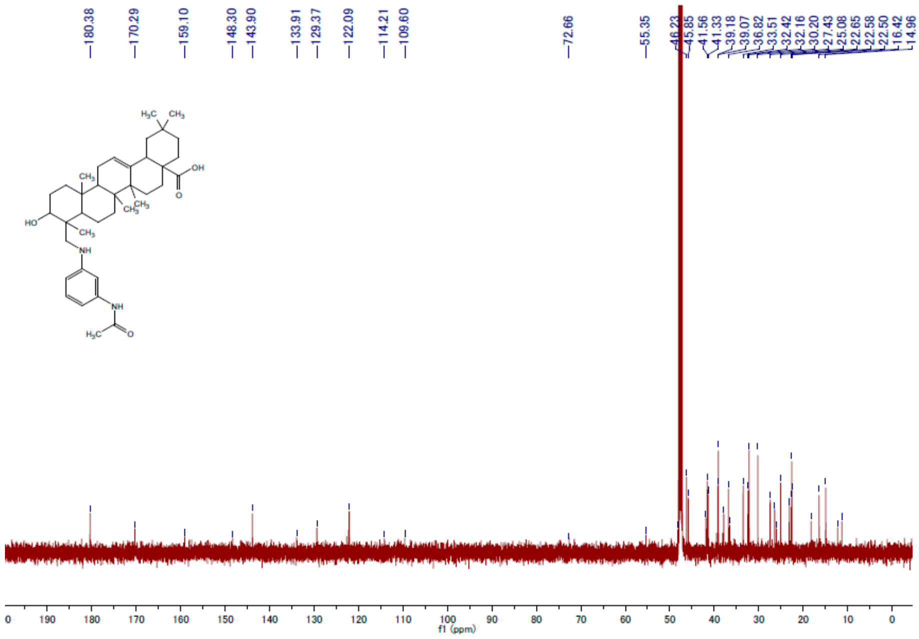

Figure S8: ^{13}C NMR spectrum of compound **13**
